# Field-based validation of a citrandarin buffer system for gene flow containment in genetically modified sweet orange trials

**DOI:** 10.3389/fbioe.2026.1929107

**Published:** 2026-10-20

**Authors:** Maria Heloisa Moreno Julião, Fabrício José Jaciani, Thais Maryana do Nascimento Guimarães, André Luis Sanches, Tatiane Maria Malara Cardamoni, Viviani Vieira Marques, Eliane Cristina Locali, Leandro Peña, Nelson Arno Wulff

**Affiliations:** 1 Biotechnology Laboratory, Research & Development Department, Fundo de Defesa da Citricultura - FUNDECITRUS, Araraquara, Brazil; 2 Institute of Chemistry, São Paulo State University UNESP, Araraquara, Brazil; 3 Instituto de Biologıa Molecular y Celular de Plantas (IBMCP), Consejo Superior de Investigaciones Cientıficas (CSIC) Universidad Politecnica de Valencia (UPV), Valencia, Spain

**Keywords:** citrandarin buffer, containment strategies, genetically modified sweet orange, Pollen-mediated gene flow, transgene

## Abstract

Containment measures to suppress the dispersion of transgenes from plots of genetically modified (GM) citrus into the progeny of surrounding non-GM citrus trees were evaluated under open-field conditions using border plants as a buffer zone. The border consisted of two plant rows each of citrandarin, selected for its superior pollination ability; Clemenules clementine, selected for its monoembryony combined with self-incompatibility and its efficiency as a pollen-mediated gene flow monitoring genotype; and Pera sweet orange, a polyembryonic commercial genotype used to estimate GM seedling frequency, arranged sequentially from the inner GM plot outward. Four assays with GM citrus plots were evaluated over two to four consecutive years for pollen flow, summing 12 seasons. Pollen compatibility among sweet orange cultivars, citrandarin, and clementine flowers was assessed using controlled crosses. Gene flow was assessed by the presence of neomycin phosphotransferase II (*npt*II) transgene in seedlings from clementine, Pera sweet orange border plants, as well as from clementine control trees located inside the plots. Trifoliate phenotype and microsatellite markers in clementine seedlings were used as indicators for paternity analysis. Transgene frequency in Pera sweet orange border plants was only 0.31%, meanwhile transgene frequency at the clementine border was lower (6.05%) than that in the controls within the plot (24.09%), working as a mitigating strategy to reduce gene flow. Overall, the superiority of citrandarin pollination and fertilization in comparison to other citrus genotypes, the distance among the genotypes indicated by the physical proximity between pollen-receptor and citrandarin, and the genetic barriers of facultative apomixis and self-compatibility in sweet orange genotypes strongly limits, but does not prevent GM pollen dispersion, favoring fertilization from citrandarin instead of the GM sweet orange genotypes into Clemenules.

## 1 Introduction

Citrus fruits represent one of the most widely cultivated fruit crops globally, and the citrus industry plays a significant role in the global economy, with orange juice as a worldwide commodity. However, in recent years, citriculture has faced severe climatic challenges that have compromised fruit production and quality, negatively impacting the entire citrus value chain (Food and Agriculture Organization of the United Nations, 2024; Fundecitrus, 2024; United States Department of Agriculture Foreign Agricultural Service, 2024), along with pests and diseases that severely affect citrus farming globally (Barbieri et al., 2023). Among these, huanglongbing is the main challenge facing citrus farming worldwide (Bassanezi et al., 2020).

In addition to the possibility of generating solutions to huanglongbing and other challenges in citriculture, biotechnology enables the development of genetically modified (GM) citrus via genetic engineering, thereby overcoming obstacles imposed by conventional breeding methods in some cases (Germanà et al., 2020). However, the coexistence of GM and non-GM citrus in the same geographical area raises issues that must be considered by scientists and regulatory agencies, including unintentional transgene flow (Hüsken et al., 2010). In plants, gene flow (GF) is mediated by pollination, seed dissemination, or vegetative propagules (Lu, 2008). In the context of the biosafety of GM crops, GF is considered a key element that can be measured from the non-GM plant hybrid progeny generated by the fertilization of GM pollen, through molecular identification of the transgene(s) or indirectly through analysis with phenotypic markers. The pollen-mediated gene flow (PMGF) is important in citrus species for measuring transgene dispersal. However, citrus seeds are not intentionally consumed by humans, although there are records of consumption and dispersal of sweet orange seeds by animals, particularly wild boar (Peris et al., 2019). Furthermore, the presence of GM zygotic embryos in seeds from non-GM plants can have implications for fruit marketing and consumer acceptance (Ellstrand et al., 2003; Ellstrand, 2014), but it does not affect juice or co-products derived from plant parts that do not contain the zygotic embryo.

Moreover, GM zygotic embryos in seeds do not represent a significant factor in seed dispersal, since sweet orange trees are highly dependent on human intervention for propagation and persistence in the environment. The lower viability of zygotic seedlings, associated with homozygous constraints, also reduces the possibilities of adventitious propagation. In addition, sweet orange fruits are exotic on the American continent, and there are practically no sympatric wild populations or relatives in citrus production areas in the world, except in Southeast Asia; this limits the potential for GM sweet orange plants, a priori, to become wild populations (Talon et al., 2020).

The particularities of the citrus reproductive system can directly influence GF. Citrus flowers are attractive to pollinating insects and are considered entomophilous, pollinated mainly by insects, especially bees (Malerbo-Souza et al., 2003; Malerbo-Souza et al., 2008; Malerbo-Souza, 2010; Malerbo-Souza, 2013; Nascimento et al., 2011). Flower morphology favors cross-pollination, and in some genotypes, self-incompatibility prevents self-fertilization. In addition, facultative apomixis results in nucellar embryo development from maternal cells of mature ovules through successive mitotic divisions, alongside zygotic embryos of sexual origin, enabling the production of progeny genetically identical to the mother plant (Cameron et al., 1957; Koltunow et al., 1996; Aleza et al., 2010).

The Brazilian National Technical Biosafety Commission (CTNBio), in Normative Resolution No. 10 (BRASIL 2013), established the use of a system of border plants around experimental areas with GM sweet orange plants. The strategy is based on the superior competitive capacity for pollen fertilization of citrandarins (superpollinators) and the low reproductive ability of sweet orange species, which together constitute barriers to GF (Pons et al., 2011; BRASIL. Comissão Técnica Nacional de Biossegurança, 2013; Sanches, 2014; Guimarães, 2021). Three types of plant borders should be used, each consisting of two crop lines, with the following non-GM genotypes: (1) a pollinator, citrandarin (*Citrus reshni* hort. ex Tanaka × *Poncirus trifoliata* [L.] Raf.); (2) a self-incompatible and monoembryonic pollen receptor (such as Clemenules clementine; *Citrus* × *clementina* hort. ex Tanaka); and (3) at least two crop lines of a sweet orange variety (*Citrus* × *sinensis* [L.] Osbeck) (BRASIL. Comissão Técnica Nacional de Biossegurança, 2013). Clemenules clementine has the sole role of allowing assessment of GF.

Herein, we evaluate the effectiveness of this plant border buffer system by assessing seedlings of Clemenules clementine (a self-incompatible, monoembryonic genotype) and sweet orange (a self-compatible, polyembryonic genotype) generated from seeds and fruits of plants subjected to open pollination in GM citrus plots. Pollen compatibility between the citrandarin source and sweet orange sources with Clemenules clementine was assessed in controlled pollinations (Pons et al., 2011). Pollen flow was measured as the ability of either citrandarin or GM and non-GM sweet orange plants, acting as open-pollinated pollen donors, to fertilize and generate seedlings in Clemenules clementine and Pera sweet orange. We expect these results to guide regulatory aspects of experimentation and the cultivation of GM citrus, given the limited likelihood of transgene flow from GM to non-GM polyembryonic genotypes, particularly to sweet orange with overlapping flowering phenotypes.

## 2 Materials and methods

### 2.1 Experimental areas and genotypes

The GF evaluations were conducted in four areas—Planned Release into the Environment—designated as experiments 1, 2, 3, and 6, established under the terms of Normative Resolution No. 10 (CTNBio), located in Ibaté, SP (experiments 1, 3, and 6; irrigated) and Santa Cruz do Rio Pardo, SP (experiment 2; rain-fed), Brazil. These areas were implanted in December 2013 (experiment 1), November 2014 (experiment 2), January 2016 (experiment 3), and December 2019 (experiment 6). Experiment 6 originated from a reformulation of adjacent experiments 1 and 3 and was implemented in the area previously occupied by border plants from experiment 1, border plants from experiment 3, and GM and non-GM (control) plants from within the bordered area. Part of experiment 3 was planted in December 2018, at the opposite end from where sampling occurred. Part of experiment 2 was planted in 2017 on the left side, also at the opposite end where sampling occurred; in 2019, a modification in the central area included the planting of several genotypes with the same gene construct, where sampling was not conducted. In all experiments, row spacing was 6.5 m, and spacing between plants within rows was 2.5 m (615 plants per hectare) (Figure 1). Plots were implanted within sweet orange farms. Additional information on area size, number of plants, and geographical coordinates is provided in Supplementary Table S1.

**FIGURE 1:**
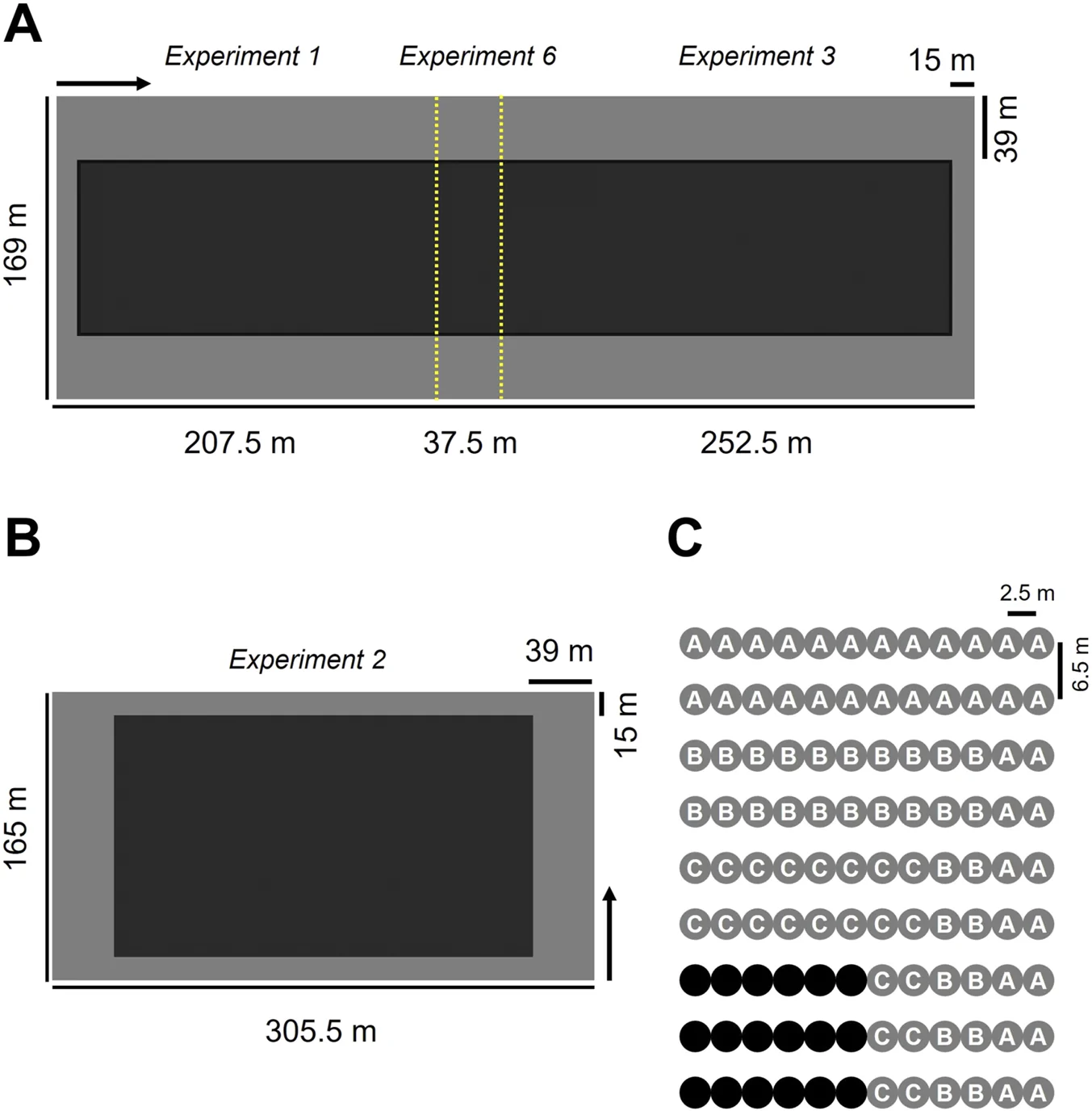
Diagram of experiments to study gene flow in genetically modified sweet orange trials conducted from 2018 to 2023 using buffer zones. Experiments 1, 3, and 6 **(A)** and experiment 2 **(B)** have a border (shown in gray) surrounding the central area containing GM and non-GM citrus (dark-gray rectangles). **(C)** Diagram of the border system according to CTNBio Normative Resolution No. 10, consisting of two rows of non-GM plants of each genotype: Pera sweet orange **(A)** Clemenules clementine **(B)** citrandarin **(C)** (letters inside gray circles). Black circles represent the central area containing GM and non-GM citrus. Arrows indicate the row orientation. Row spacing was 6.5 m and tree spacing was 2.5 m. Drawn not to scale.

The climate classification of the locations is CWA (high altitude tropical with dry winter) in Ibaté and CFA (subtropical climate) in Santa Cruz do Rio Pardo (Köppen). The predominant biome in Ibaté is the Cerrado, whereas in Santa Cruz do Rio Pardo, it is the Atlantic Forest (IBGE, 2024). Air temperature, relative humidity, and rainfall were recorded at a meteorological station (MS) located on the farm of experiment 2 from July 2018 to July 2020, and at the closest MS to experiments 1, 3, and 6, approximately 5 km from the trial area, from July 2019 to July 2023. Monthly averages of temperature, relative humidity, and rainfall are shown in Supplementary Figure S1.

The rootstock genotypes used were Swingle citrumelo (*Citrus paradisi* MacFad. × *Poncirus trifoliata* [L.] Raf.) in experiments 1, 3, and 6 and Rangpur lime (*Citrus* × *limonia* Osbeck) in experiment 2. The biosecurity border plants in experiments 1, 3 and 6 were as follows: Pera sweet orange (non-GM, polyembryonic, self-compatible), Clemenules clementine (non-GM, monoembryonic, self-incompatible), and citrandarin (*Citrus reshni* hort. ex Tanaka × *Poncirus trifoliata* [L.] Raf., a non-GM superpollinator). The citrandarin accessions comprised accession 1600 from the Centro de Citricultura Sylvio Moreira (experiment 1) and accession H3 from Instituto Valenciano de Investigaciones Agrarias (experiments 2, 3 and 6) (Pons et al., 2011). GM plants incorporate the *npt*II gene cassette, which encodes neomycin phosphotransferase type II and confers resistance to aminoglycoside antibiotics; its expression is controlled by the constitutive CaMV35S promoter (from *Cauliflower mosaic virus*) or the NOS (nopaline synthase) promoter from *Agrobacterium tumefaciens* (Pons et al., 2014; Santos, 2019; Alquézar et al., 2021). This characteristic was used to monitor GF from GM sweet orange genotypes using molecular detection techniques. Information on GM scion genotypes is available upon request to the corresponding author.

Pollen from Pera, Hamlin, Valencia, and Pineapple sweet oranges cultivars (*Citrus* × *sinensis* [L.] Osbeck), Citrandarin 1600 (*Citrus reshni* hort. ex Tanaka × *Poncirus trifoliata* [L.] Raf. Rubidoux) was collected from commercial farms or seed banks located in the central region of São Paulo State. Hand-controlled pollinations were carried out on clementine flowers over two seasons: one at the Centro de Citricultura “Sylvio Moreira” (IAC) in Cordeirópolis (2011/2012) and the other on a farm in Botucatu, SP (2013/2014).

### 2.2 Controlled crosses for pollen compatibility analysis

Pollen grains were collected from sweet oranges cultivar (Pera, Hamlin, Valencia, and Pineapple) and Citrandarin 1600 from flowers before anthesis, then dried, counted, and stored as previously described (Pons et al., 2011; Sanches, 2014). Single or mixed pollinations were carried out on hand-opened Clemenules clementine flowers using a pencil; the open stigma were then covered with paper bags, and fruits were collected near maturation to assess fruit set, seed number, phenotypic expression of the trifoliate trait, and microsatellite profiles of monofoliate seedling (Sanches, 2014).

### 2.3 Border system

The border system in all experiments is arranged as established in Normative Resolution No. 10 (CTNBio) and consists of two rows for each genotype: (1) citrandarin, a superpollinator genotype capable of effectively competing for fertilization with pollen from sweet orange trees; (2) Clemenules clementine, a monoembryonic and self-incompatible genotype; and (3) Pera sweet orange, a polyembryonic genotype (BRASIL. Comissão Técnica Nacional de Biossegurança, 2013). The first and second rows of border plants of each genotype surrounding the GM area are named internal border and external border, respectively (Figure 1).

### 2.4 Fruit collection and seed extraction

Gene flow evaluations were conducted between 2018 and 2023, 3–4 years after the planting of each experiment, when flowering and fruit set are regular for commercial citrus groves and for citrus varieties generated using mature explants for genetic transformation. The 2018 data were obtained previously (Guimarães, 2021). Plant sampling was completely randomized (Figures 2, 3). The same plants sampled in the first assessment of each experiment were selected for sampling in all subsequent seasons/years. However, the number of sampled plants varied between years due to the eradication of plants with greening disease (eradication rates reached up to 54.9% in Pera sweet orange in experiment 1; Supplementary Table S2), which also reduced the sources of non-GM pollen throughout the years and altered the proportion of GM and non-GM plants in the experimental areas.

**FIGURE 2:**
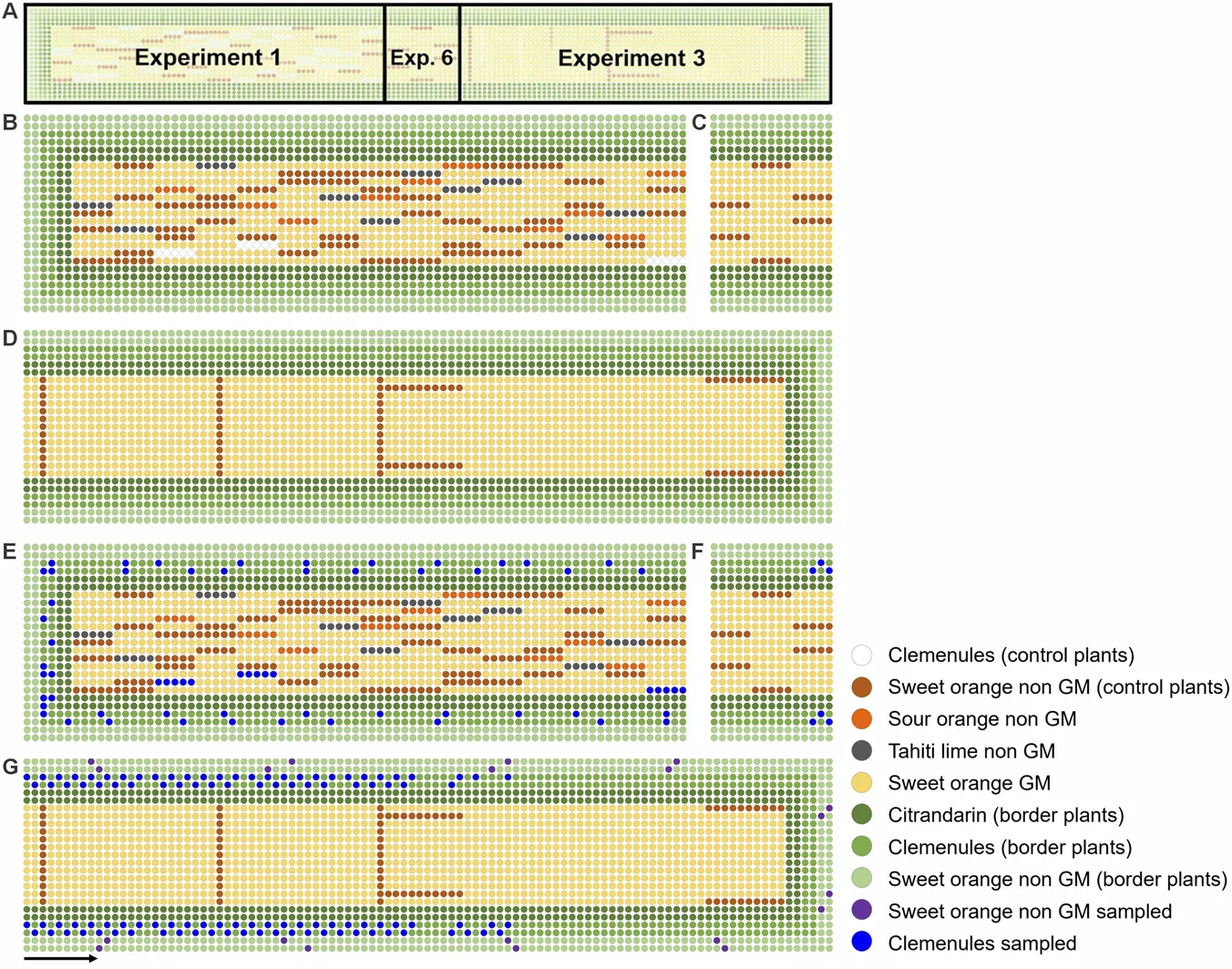
Diagram of experiments to study gene flow in genetically modified sweet orange trials conducted from 2018 to 2023 using buffer zones. Experiments 1, 3, and 6 **(A)** as set up in field conditions. Citrus genotypes for each tree position: **(B)** (experiment 1); **(C)** (experiment 6); **(D)** (experiment 3), according to the legend. Citrus genotypes for each tree position, indicating citrus trees with sampled fruits **(E,F,G,)** respectively experiments 1, 6 and 3. Tree genotype according to the legend. The border system following CTNBio Normative Resolution No. 10, consists of two rows of non-GM plants of each genotype: sweet orange, Clemenules clementine, and citrandarin (green circles, see legend). The central area contains GM and non-GM citrus (controls). The arrow indicates the row orientation. Row spacing was 6.5 m and tree spacing was 2.5 m.

**FIGURE 3:**
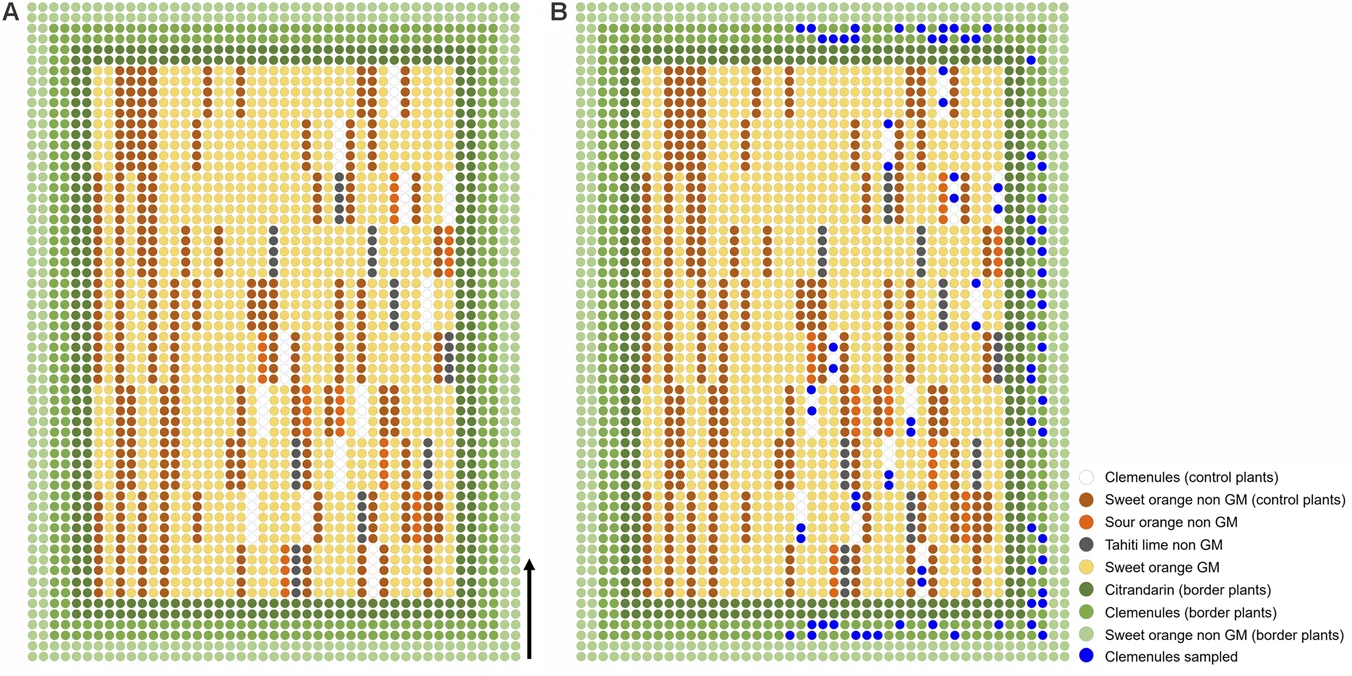
Diagram of experiments to study gene flow in genetically modified sweet orange trials conducted from 2018 to 2020 using buffer zones. Experiment 2 **(A)** as set up in field conditions. Citrus genotypes for each tree position according to the legend. **(B)** Citrus trees genotypes with sampled fruits. The border system following CTNBio Normative Resolution No. 10, consists of two rows of non-GM plants of each genotype: sweet orange, Clemenules clementine, and citrandarin (green circles, see legend). The central area contains GM and non-GM citrus (controls). The arrow indicates the row orientation. Row spacing was 6.5 m and tree spacing was 2.5 m.

The control Clemenules clementine parcels evaluated in experiment 1 were located within the plot in rows at distances of 19.5, 13.0, or 6.5 m from the internal citrandarin border (Figure 2), whereas in experiment 2, sampled plants were located at distances ranging from 2.5 to 104 m from the internal citrandarin border (Figure 3). Between 25 and 100 open-pollinated fruits were collected per plant. After seed extraction, asepsis was carried out on all the seeds, followed by sowing. No internal control parcels of clementine were included in experiments 3 and 6.

Seeds from clementine and Pera sweet orange, obtained from both controlled and open-pollination assays (Figures 2, 3), were collected, counted, dried, and sown in coconut fiber under nursery conditions.

### 2.5 Phenotypic analysis

Phenotypic analysis was conducted between 90 and 120 days after sowing Clemenules clementine seeds in the greenhouse. Seedlings were classified according to leaf morphology as trifoliolate or monofoliate (Supplementary Figure S2). The trifoliate characteristic, a dominant trait over the monofoliate trait (Soares Filho et al., 2000), is exclusively present in citrandarin plants across all experimental areas (a non-GM genotype). This feature allows identification of the citrandarin genotype as the parent of trifoliate progeny in open-pollinated experiments and controlled pollinated assays.

### 2.6 Molecular analysis

A leaf tissue disc (6 mm in diameter) was collected from each monofoliate progeny seedling for DNA extraction (Murray and Thompson, 1980). A 6 mm diameter stainless steel ball and 400 µL of extraction buffer (CTAB 2%; NaCl 1.4M; PVP 2% [10,000]; EDTA 0.5M, pH 8; Tris-HCl 1M, pH 8; β-mercaptoethanol 0.2%) were added to each microtube. The samples were ground in an automatic grinder (TissueLyser II, Qiagen) for 30 s at 30 Hz and kept in a water bath at 65 °C for 30 min. Then 400 μL of chloroform was added and homogenized by vortexing for 30 s. The tubes were centrifuged at 15,294 *g* at 23 °C for 5 min, followed by recovery of the supernatant (200 μL) and addition of isopropanol (120 μL). The samples were incubated at –20 °C for at least 30 min and then centrifuged at 15,294 *g* for 15 min. The supernatant was discarded, and 1 mL of 70% ethanol was added to the precipitate, followed by centrifugation for 10 min at 15,294 *g*. The supernatant was discarded, and the precipitate was vacuum-dried (Concentrator Plus, Eppendorf) for 7 min and resuspended in 40 μL of ultrapure water.

Positive controls (DNA extracted from confirmed transgenic plant tissue) and negative controls (DNA from non-GM control plants and no-template controls, NTC) were included in every PCR run. Primer sensitivity was previously validated to detect target transgenic DNA from total genomic DNA. Amplifications yielding ambiguous or weak bands were re-extracted and re-analyzed in duplicate to avoid false negatives or false positives.

### 2.7 Detection of the selective marker for genetically modified progenies

PCR reactions were carried out to detect the *npt*II gene using specific primers (Ghorbel et al., 2000; Supplementary Table S3). PCR conditions were as follows: 1X PCR buffer, 0.15 mM deoxyribonucleotides, 1.5 mM magnesium chloride, 2 units of Taq DNA polymerase (Invitrogen), 0.3 µM of each primer, 1 µL of genomic DNA (approximately 100 ng), and ultrapure water to a final volume of 20 µL.

The amplification cycles consisted of an initial denaturation at 94 °C for 3 min, followed by 35 cycles of denaturation at 94 °C for 30 s, annealing at 62 °C for 30 s, and extension at 72 °C for 45 s, with a final extension at 72 °C for 10 min. The PCR products were resolved by 1.0% agarose gel electrophoresis in TAE 1X buffer (40 mM Tris-HCl, 40 mM acetic acid, 1 mM EDTA), using a 100 bp molecular mass marker (Invitrogen) as a ladder. The gels were stained with ethidium bromide (0.1 µg/mL) and visualized using a UV transilluminator (Sambrook and Russell, 2006).

### 2.8 Microsatellite analysis for paternity assignment of Clemenules clementine monofoliate phenotype

Paternity assignment using microsatellite marker analysis was performed on 20 progeny samples per plant, randomly selected from those showing negative PCR results for the transgene and exhibiting monofoliate phenotype, derived from the open-pollination assays. Triplex PCR reactions were optimized, and samples were analyzed using six pairs of primers, with forward primers labeled with the fluorescent dyes TAMRA, FAM, or HEX (Tables 1 and 2).

**TABLE 1:** Specifications of primers used in PCR reactions for genotyping with microsatellite molecular markers in open pollination paternity analysis.

Marker name	Motif	Size (pb)	Sequence (forward/reverse)	References
mCrCIR03C08	—	200–225	CAGAGACAGCCAAGAGA GCTTCTTACATTCCTCAAA	Cuenca et al. (2011)
mCrCIR01F08a	(GAAT)_6_	110–136	ATGAGCTAAAGAGAAGAGG GGACTCAACACAACACAA	Froelicher et al. (2008)
Ci07E06	(GA)_8_	216–245	AATAAACGCCCACCTGAGAC CAGTTGTTAAAAGGGAAGAATGAA	
mCrCIR07E05	(GA)_9_	110–130	GGAGAACAAAACACAATG ATCTTTCGGACAATCTT	
Ci01C07	(CT)_10_	240–265	GTCACTCACTCTCGCTCTTG TTGCTAGCTGCTTTAACTTT	
Ci02B07	(GA)_14_	160–175	CAGCTCAACATGAAAGG TTGGAGAACAGGATGG	

**TABLE 2:** Expected allele sizes (bp) for microsatellite markers (SSR) in potential citrus pollen donor genotypes.

Genotype/SSR	Ci03C08	Ci01F08a	Ci07E06	Ci07E05	Ci01C07	Ci02B07
Clemenules	204/223	115	227	110/112	243	163/165
Sweet oranges	204/209	115/135	227/242	110/114	262	161/165
Citrandarin	212/220	111/115	227/238	112/122	243/258	170

PCR reactions were carried out in a final volume of 20 µL containing 1× PCR buffer, 0.2 mM of each dNTP, 5 mM MgCl_2_, 0.5 µM of each primer, 1 U of Taq DNA polymerase (Invitrogen), 1 µL of genomic DNA (approximately 100 ng), and ultrapure water to complete the volume. The amplification program consisted of an initial denaturation at 94 °C for 5 min, followed by 40 cycles of denaturation at 94 °C for 30 s, annealing at 60 °C for 1 min, and extension at 72 °C for 30 s, with a final extension at 72 °C for 45 min.

From the final PCR product, 1 µL was recovered and diluted with 9 µL of water. From this dilution, a 1 µL aliquot was mixed with 0.2 µL of GeneScan™ 500 ROX™ Size Standard and 8.8 µL of formamide (Applied Biosystems). Fragment separation was performed using an ABI 3730XL DNA Analyzer (Applied Biosystems®, Foster City, CA, United States), and data were analyzed using GeneMapperTM software version 6 (Thermo Fisher Scientific Inc). Samples for which paternity could not be assigned were considered indeterminate.

### 2.9 Data analysis

The number of seeds per fruit from controlled pollination data was transformed into log for statistical analysis using Bartlett’s test for homogeneity of variance.

The mean GF per plant was calculated by dividing the number of samples testing positive for the transgene in each plant by the total number of germinated progeny seeds from that plant, then multiplying the result by 100 to obtain a percentage. This GF percentage per plant was subsequently used to calculate the mean GF for each border, treatment (rows for control plants), and season, as well as to compute overall means across borders, treatments, and seasons. The means were not averaged across groups.

The response variable (GF) was transformed using the natural logarithm plus one to meet the assumptions of normality and homogeneity of variance, when necessary. GF was first calculated independently for each recipient tree as the percentage of its analyzed progeny testing positive for *npt*II, and these tree-level values were subsequently used in the statistical analyses. Therefore, variation in the number of progenies among trees represents unequal subsampling intensity within experimental units rather than unequal biological replication. Moreover, such variation in progeny number is biologically expected, as both the number of fruits produced per tree and the number of seeds per fruit naturally vary among individual trees and across seasons/years. Analysis of variance (ANOVA) was performed on the transgene flow data, followed by comparison of means using Tukey test at a 5% significance level.

The relationship between GF and the distance from recipient Clemenules clementine trees to citrandarin was investigated using linear regression, with GF as the response variable and distance as the independent variable. The analyses were conducted using R and Excel software (R Core Team, 2021; Microsoft Corporation, 2024).

## 3 Results

### 3.1 Pollen compatibility and competitive pollination between citrandarin and sweet orange in Clemenules clementine

Pollen grains from Pera, Valencia, Hamlin, Pineapple sweet oranges cultivars, as well as Citrandarin 1600, were compatible with Clemenules clementine, as measured by fruit set and seed production (Table 3), showing slight variation with genotype and season (Sanches, 2014). Paternity analysis of clementine pollinations from the 2011/2012 season indicated superior pollination performance of Citrandarin 1600 compared with Pera and Pineapple sweet orange pollen when mixed pollinations were used, as measured by the presence of the trifoliate phenotype and genotypic markers in the resulting progeny (Supplementary Table S4) (Sanches, 2014).

**TABLE 3:** Average number of seeds in Clemenules clementine fruits obtained from controlled pollination under field conditions.

Treatments/Season	2011/2012		2013/2014	
	Average ± SE	n	Average ± SE	n
Citrandarin 1660	14.76 ± 1.23 a	46	14.34 ± 0.45 a	91
Pera sweet orange	12.71 ± 2.53 a	7	16.61 ± 0.40 a	240
Valencia sweet orange	Nd		11.5 ± 0.50 b	164
Hamlin sweet orange	16.4 ± 3.02 a	10	11.13 ± 0.48 b	102
Pineapple sweet orange	9.75 ± 1.23 a	24	16.35 ± 0.62 a	104
Pera + citrandarin	9.46 ± 0.98 a	57	15.09 ± 0.34 a	269
Pineapple + citrandarin	11.06 ± 0.86 a	68	Nd	
Valencia + citrandarin	Nd		14.93 ± 0.32 a	227

n, number of Clemenules fruits per treatment; Nd, not determined.

Season 2011/2012 = F (5, 206) = 3.25** (p < 0.01) CV% = 38.5; χ^2^ < χ^2^ (5%), indicating that variances are homogeneous (p > 0.005).

Season 2013/2014 = F (6, 1190) = 21.55 (p < 0.01) CV% = 19.03; χ^2^ > χ^2^ (1%), indicating that variances are non-homogeneous (p > 0.01).

### 3.2 Transgene flow in border plants

In clementine border plants, sampling resulted in the collection of approximately 29,000 fruits for GF assessment, of which the percentage of seedless fruits ranged from 16% to 64%. The overall GF, calculated from 23,148 seedlings analyzed for the presence of *npt*II marker, was 6.05%, based on the average GF of all sampled Clemenules clementine border plants (Table 4). The total number of plants sampled, fruits collected, and seeds extracted per year is detailed in Supplementary Table S5. Figure 4 shows an aerial view of part of Experiment 1, highlighting the border rows surrounding the entire field trial and the spatial arrangement of the internal rows containing experimental parcels, used to assess pollen-mediated gene flow.

**TABLE 4:** Transgene flow (%) in Clemenules clementine border plants.

Experiment	Season	Seedlings^1^		Gene flow (%)		Average^2^
		Internal	External	Internal	External	
1	2018/19	2,836	Na	12.06	Na	12.06
	2019/20	1,991	1,591	3.16	17.43	10.54
	2020/21	345	683	2.62	3.53	3.18
	2021/22	1,151	1,651	4.92	1.89	3.34
2	2019/20	1,242	558	13.15	19.26	15.79
	2020/21	800	916	2.79	1.48	2.08
3	2020/21	1,158	1,223	1.72	3.42	2.59
	2021/22	1,125	1,484	4.84	4.30	4.55
	2022/23	2,205	1,808	3.36	8.00	5.89
6	2020/21	29	40	13.6	26.4	21.3
	2021/22	114	47	6.34	2.84	5.42
	2022/23	106	45	3.89	18.94	7.65

1 Internal and external are the borders in relation to the GM, plots; Na = Not assessed;

2 Average transgene flow in the season.

**FIGURE 4:**
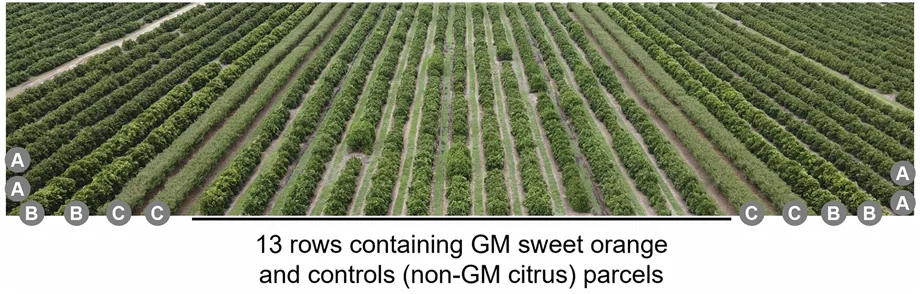
Aerial picture of the field trial encompassing part of experiment 1, taken 7 years and 4 months after planting. Border system according to CTNBio Normative Resolution No. 10, consisting of two rows of non-GM trees of each genotype (gray circles) labeled **(A)** for sweet orange, **(B)** for Clemenules clementine, and **(C)** for citrandarin. Border trees surround the whole block and the 13 internal rows contain experimental parcels. Row spacing was 6.5 m and tree spacing was 2.5 m.

In the Pera sweet orange border, a total of 900 fruits were collected over two consecutive seasons, yielding 2,996 seeds; this corresponded to an average of 2.2 (2021/22) and 4.0 (2022/23) seeds per fruit, and the percentage of seedless fruits ranged from 8.0% to 60.0% (2021/22) and from 0.0% to 20.0% (2022/23) per plant. Seed germination averaged 76%, and 32.02% of the seeds produced between two and five seedlings, which were considered polyembryonic (Supplementary Table S6).

Transgene flow in Pera sweet orange border plants was 0.31%, based on the average GF of all sampled border plants, fruits, and seeds, as determined by the presence of the transgene in seedlings grown under greenhouse conditions. There was no statistically significant difference between inner and outer borders (F = 0.0790; p = 0.7802) or between the years evaluated (F = 3.2222; p = 0.0810) (Table 5).

**TABLE 5:** Gene flow in Pera sweet orange border plants, analyzed based on the presence of seedlings carrying the neomycin phosphotransferase II transgene used as a molecular marker.

Season	Seedlings^1^		Gene flow (%)		Average
	Internal	External	Internal	External	
2021/2022	319	959	0.00 ± 0.00	0.21 ± 0.21	0.11 ± 0.11
2022/2023	1947	451	0.58 ± 0.27	0.44 ± 0.44	0.51 ± 0.25
Average			0.29 ± 0.15	0.33 ± 0.24	​

1 Internal and external are the borders in relation to the GM, plots; seedling evaluations were carried out in mono- and poly-embryonic seeds, resulting in more seedlings than the number of germinated seeds.

### 3.3 Transgene flow in control plants

Approximately 7,000 fruits were collected from clementine control plants located in parcels within the buffer zone, resulting in 12,535 extracted seeds. The number of plants, fruits, and seeds evaluated per year is detailed in Supplementary Table S7. The GF was detected in progeny from 103 out of 106 clementine control plants sampled, corresponding to a GF rate of 23.59% across experiments 1 and 2 (Table 6).

**TABLE 6:** Transgene flow (%) in Clemenules clementine control plants located within the citrandarin border, without buffer zones between GM donor plants and recipient Clemenules clementine trees.

Experiment	Nearest citrandarin distance (meters)	Season				Average
		2017/18	2018/19	2019/20	2020/21	
1	19.5	22.28	36.74	38.07	34.60	32.92
	13.0	22.90	19.91	25.72	29.33	24.61
	6.5	9.97	18.23	5.65	17.23	12.89
2	2.5–123.5	Na	25.48	20.34	Na	23.59

Na = not assessed.

A positive linear relationship was found between transgene flow and the distance of recipient plants from the citrandarin border (p = 0.008). Although the strength of this linear relationship varied between years, as indicated by variation in correlation coefficients (r = 0.179–0.687) and significance values (p = 0.006–0.2), transgene flow tended to increase as recipient plants were located farther from the citrandarin border, which was also spatially related to the distribution of GM pollen sources within the experimental area (Figure 5).

**FIGURE 5:**
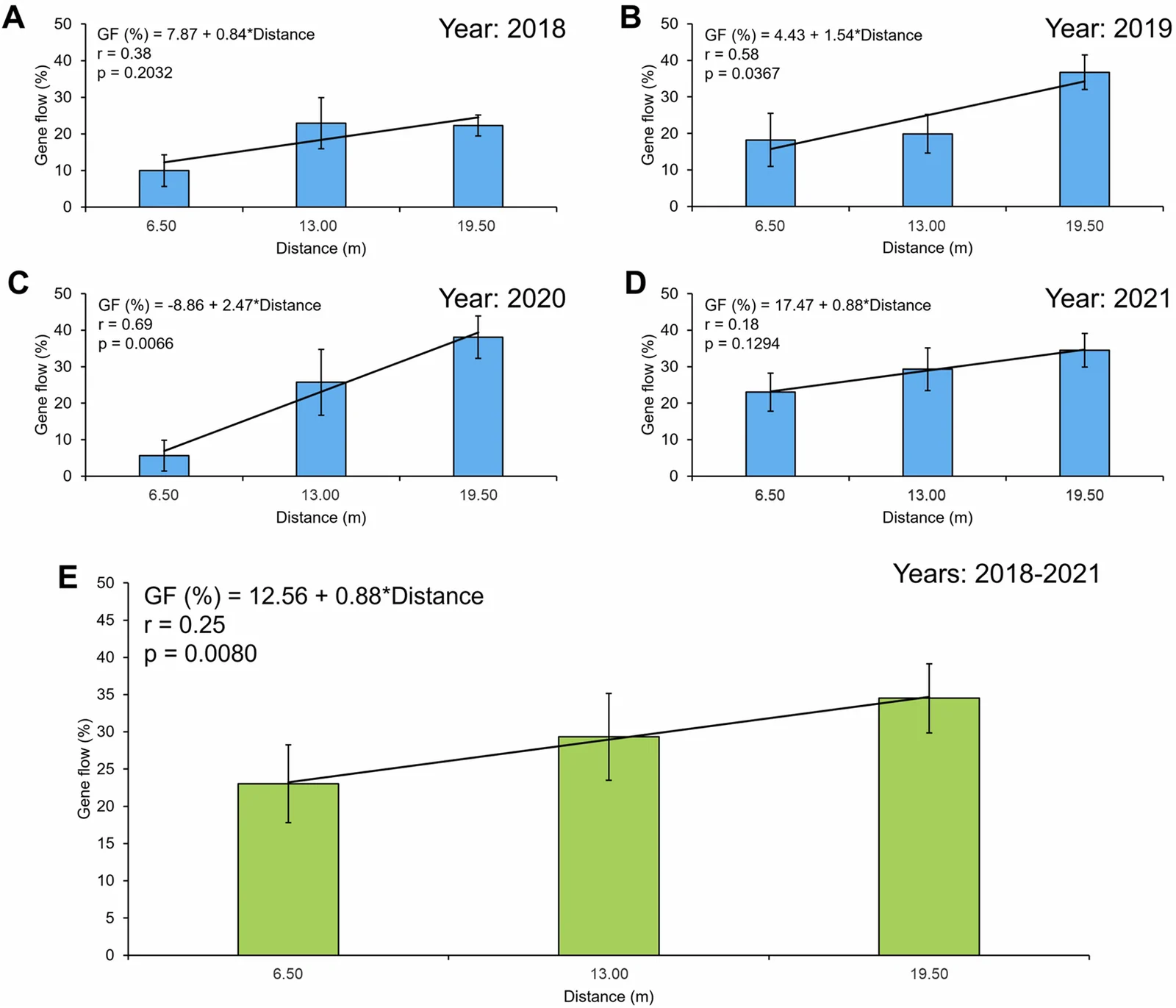
Gene flow (%) measured as the presence of *npt*II in progeny of Clemenules clementine, resulting from pollination by genetically modified donor sweet orange plants, located within the border system, in relation to the distance (meters between rows) between Clemenules clementine and citrandarin super-pollen donor plants, for **(A)** 2018, **(B)** 2019, **(C)** 2020, **(D)** 2021, and **(E)** 2018–2021.

### 3.4 Transgene flow from genetically modified sweet orange

Considering the average GF per genotype (Clemenules clementine and Pera sweet orange) and plant position (border and control) across all the experiments and years, plant position significantly influenced GF (F = 318.36; p < 0.0001), with border plants showing lower GF values than control plants. Genotype also significantly affected GF (F = 5.35; p = 0.0208), with sweet orange showing lower GF levels than Clemenules clementine (Table 7).

**TABLE 7:** Gene flow data for Clemenules clementine and Pera sweet orange plants from the border and control treatments: total number of plants sampled, germinated seedlings, seedlings positive for the transgene (*npt*II), gene flow per plant (minimum and maximum), and average gene flow per genotype and treatment across experiments 1, 2, 3, and 6 over all years evaluated.

Genotype (location)	Total plants sampled	Seedlings		Gene flow per plant (%)		Gene flow average (%)
		Total evaluated	Positive for the transgene	Min	Max	
Clemenules						
Border	559	23,148	1,656	0.0	67.24	6.05 ± 9.79
Control	93	6,201	1,245	0.0	65.02	24.09 ± 14.12
Sweet orange						
Border	40	3,676	15	0.0	4.40	0.31 ± 8.30

## 4 Discussion

Sweet oranges cultivars are highly similar in terms of genetic relatedness, and Pera, Valencia, and Hamlin are among the most widely cultivated for orange juice production. Controlled pollination experiments using Clemenules clementine as the compatible recipient parent showed that these genotypes had similar abilities to produce seeds. A similar pattern was observed for Citrandarin 1600, which however, showed a higher ability to compete for fertilization when mixed pollination with sweet orange pollen was used. The preliminary data support the use of Citrandarin and Clemenules clementine as a border system to mitigate and monitor the gene flow from genetically modified sweet orange genotypes under open-field conditions where bees, dispersing pollen preferentially to the closest trees, are the primary pollinators (Malerbo-Souza et al., 2003; Malerbo-Souza et al., 2008; Malerbo-Souza, 2010; Malerbo-Souza, 2013; Nascimento et al., 2011; Pons et al., 2011).

### 4.1 Transgene flow from genetically modified sweet orange

In most of the years we evaluated GF, intense climatic variation compromised global sweet orange production (Fundecitrus, 2024; United States Department of Agriculture Foreign Agricultural Service, 2024; United States Department of Agriculture Foreign Agricultural Service, 2018; United States Department of Agriculture Foreign Agricultural Service, 2019; United States Department of Agriculture Foreign Agricultural Service, 2020; United States Department of Agriculture, 2023). Climatic factors can influence photosynthetic enzyme activity, the availability of flowers, pollen, and nectar, pollen viability, and ovule fertility (Spiegel-Roy and Goldschmidt, 1996), as well as bee foraging patterns (Malerbo-Souza, 2013; Nascimento et al., 2011; Spiegel-Roy and Goldschmidt, 1996; Szabo, 1980). Heat waves can cause premature fruit drop, thereby reducing fruit set. These variables may have contributed to the variation observed in GF values between experiments and years, as climate factors act on multiple stages of this process. In addition, irrigation can affect citrus plant development and was present only in experiments 1, 3, and 6, but not in experiment 2.

Transgene flow was previously assessed in GM citrus (Pineapple sweet orange [*Citrus* × *sinensis* (L.) Osbeck], Troyer citrange [*C. sinensis* (L.) Osbeck *× Poncirus trifoliata* (L.) Raf.], and Mexican lime [*Citrus × aurantiifolia* Swingle]) in Valencia, Spain. The experimental area consisted of 130 adult plants, with 48 GM plants in a central plot and 82 non-GM plants (Clemenules) surrounding the area, totaling 1,638 m^2^ and a ratio of two non-GM plants for each GM plant. The GF in clementine ranged from 0.17% to 2.86% over 7 years of evaluation (Pons et al., 2011).

Climate and the presence and abundance of pollinators differ between Brazilian and European citrus-growing regions. Water stress may be the main inducer of flowering in semi-tropical conditions, whereas lower winter temperatures are the primary flowering inducer in original citrus habitats of Southeast Asia and in Spain. The duration of flowering depends on prevailing temperatures, with warmer climates (such as those observed during this study) resulting in concomitant flowering, petal fall, and fruiting (United States Department of Agriculture Foreign Agricultural Service, 2019). In this study, temperature at the experiment’s locations (central and southwestern regions of the state of São Paulo) showed less seasonal variation than the water regime, which is the main factor inducing flowering. In contrast, low temperatures are the main inducer of flowering in Europe and in the Mediterranean climate where previous experiments were conducted (Pons et al., 2011).

Moreover, the distance between the pollen-recipient plants and GM donor plants influenced the extent of GF, with transgene flow being lower in border plants than in control plants located within the border. The physical distance between pollen sources and recipient plants is a key factor determining the frequency and maximum dispersal distance of GF in plants that are primarily entomophilous (De Jong et al., 2005), such as citrus. In several plant species, the frequency of gene flow decreases as the distance from the pollen source to the recipient plant increases (Free, 1960; Degani et al., 1990; Jackson and Futch, 1997; Wallace et al., 2002; García et al., 2005). Furthermore, in response to plant spacing, individual *A. mellifera* bees can alter movement patterns and exhibit biased foraging behavior, showing a preference for one plant over another (Morris, 1993). Additionally, we verified a positive linear association between transgene flow and the distance between clementine control plants and citrandarin border plants, with transgene flow increasing as the distance from citrandarin increased. This association varied over the 4 years, highlighting the influence of multiple factors on transgene flow to recipient plants, including contributions from the citrandarin donor. These data also highlight the superior pollen efficiency and compatibility of citrandarin including spatial arrangement of donor trees, annual environmental variation, changes in pollen-source composition caused by HLB eradication which, in addition to reducing the number of recipient plants available for assessment, the removal of trees may have altered the relative abundance and spatial distribution of pollen sources, both transgenic and non-transgenic. Such changes may have contributed to differences in competition for pollen between flowering seasons and, consequently, to the observed annual variation in gene flow frequencies caused by the eradication of HLB. Together with the controlled pollination assays, these observations support competitive pollination by citrandarin as an important contributing mechanism.

The molecular analysis tools used in this study like conventional PCR allowed robust, binary (positive or negative) screening of thousands of individual seedlings in this multi-year field study. Future biosafety and environmental monitoring could benefit from recent advances in systems for high-throughput nucleic acid evaluation (Chi et al., 2025). Integrating these next-generation diagnostic tools into GM citrus oversight could further enhance transgene surveillance efficiency in commercial settings.

### 4.2 Sweet orange exhibited reduced transgene flow compared to Clemenules

Pera sweet orange border plants were located 32.5 or 39.0 m from the nearest GM parcels, whereas Clemenules plants were 19.5 or 26.0 m away, which may have contributed to lower GF in sweet orange. Moreover, the transgene was detected in progeny from 97% of clementine control plants.

In addition to the distance between pollen-receiving plants and GM plants, intrinsic factors of sweet orange genotypes may also have contributed to the lower GF. In sweet orange, self-pollination occurs, whereby self-pollen can compete for fertilization with viable pollen from other plants (including GM pollen) and promote seed formation. In contrast, Clemenules clementine is self-incompatible, and its own pollen does not fertilize ovules (Reuther et al., 1978; Azevedo et al., 2013); consequently, there is no competition with exogenous pollen for fertilization.

The impact of the lack of competition between self-pollen and exogenous pollen on GF in clementine can be compared with GF observed in female and hermaphrodite plants of *Carica papaya* (papaya). In hermaphrodite papaya plants, the floral structure promotes self-pollination (Gonsalves et al., 2012; Manshardt et al., 2016), resulting in a GF of 1.3%, whereas in embryos of female plants, where self-pollination does not occur, GF was around 50 times higher (67.4%) (Azevedo et al., 2013). GF was also evaluated in papaya plants that were apparently established and growing without human assistance at various distances from commercial GM papaya cultivation areas. In fruits from hermaphrodite plants, no GM material was detected, whereas 16% of fruits from female plants contained at least one GM seed (Manshardt et al., 2016). The higher GF observed in female compared with hermaphrodite papaya plants indicates that the capacity for self-pollination, as in sweet orange, can limit PMGF, in contrast to the absence of self-pollination in Clemenules clementine.

Furthermore, most analyzed sweet orange seeds produced two or more seedlings and were classified as polyembryonic. In polyembryonic sweet orange cultivars, facultative apomixis leads to intense intra-seed competition between maternal nucellar embryos and the sexual zygotic embryo. This competition can suppress the survival or germination of zygotic embryos harboring the transgene, potentially leading to a lower apparent frequency of detected transgenic seedlings.

Competition for nutrients among embryos in polyembryonic seeds can favor the germination and development of non-GM nucellar embryos over GM embryos generated through transgene pollen flow, thereby reducing the likelihood of adventitious survival of GM sweet orange seedlings. Survival of sweet orange seedlings without human intervention under natural conditions is of limited importance, and no natural populations of sweet orange have been established worldwide to our knowledge. Apomixis, a genetic barrier that suppresses PMGF in the obligate apomictic species *Paspalum notatum* (Sandhu et al., 2010), may also mitigate GF in the facultatively apomictic sweet orange. This characteristic is absent in monoembryonic Clemenules clementine.

Thus, characteristics of the reproductive biology of sweet orange genotypes (self-compatibility and polyembryony) together with the spatial separation between GM plants and non-GM border plants and the competitive pollination capacity of citrandarin, represent a set of strategies to contribute collectively to the mitigation of gene flow in GM sweet orange.

Moreover, flowering synchrony among plants influences survival, fecundity, and GF (Reuther et al., 1978). Floral synchrony promotes flower availability, concurrent viability of pollen grains and stigmas, and synchronization of pollen and pistil development post-pollination, as the pistil is receptive for a relatively short period (Herrero, 2003). In contrast to other citrus species with more defined and concentrated flowering periods, Pera sweet orange exhibits multiple flowering events throughout the year, promoting greater flower availability and pollination opportunities within the same plant population compared with other varieties in the experiments, thereby reducing pollinator movement between genotypes and concentrating visits on plants of the same genotype, particularly Pera sweet orange trees. Results reported here show that transgene flow between polyembryonic genotypes is limited by distance, in addition to reproductive constraints imposed by facultative apomixis, and can be further reduced in the presence of citrandarin buffer, a genotype with high pollination efficiency.

## 5 Conclusion

The results obtained over two to four consecutive years (12 flowering seasons) indicate that the evaluated buffer zone system containing citrandarin was associated with a substantial reduction in pollen-mediated gene flow from genetically modified sweet orange under the field conditions studied. Furthermore, the facultative apomixis and self-compatibility in sweet orange genotypes were important factors in suppressing GF compared with Clemenules clementine. Gene flow in citrus plants is a complex process involving multiple interacting factors, including reproductive biology of the recipient genotypes (such as self-incompatibility and flowering synchrony), site, year, climatic conditions and the distance between recipient plants and GM pollen sources or citrandarin plants, which can reduce the occurrence of transgene flow (Pons et al., 2011). In Brazil, this buffer zone system, established by Normative Resolution No. 10 from CTNBio, was revoked by Normative Resolution No. 30 of 16/09/2020, which mandates the use of three or more rows of non-GM polyembryonic genotypes around GM citrus areas, preferably citrandarin (BRASIL. Comissão Técnica Nacional de Biossegurança, 2020). This raises concerns, as it represents a new strategy whose effectiveness in reducing pollen-mediated gene flow has not been fully validated when it allows the option of not using citrandarin as a border. Here, we present evidence that the use of citrandarin as a super-pollen donor is associated with reduced gene flow to polyembryonic sweet orange under the evaluated experimental conditions.

## Data Availability

The original contributions presented in the study are included in the article/Supplementary Material, further inquiries can be directed to the corresponding author.
