# Supplementary material for: Field-based validation of a citrandarin buffer system for gene flow containment in genetically modified sweet orange trials
Source: Front Bioeng Biotechnol. 2026 Oct 20;14:1929107. doi: 10.3389/fbioe.2026.1929107 (PMC13727559; doi:10.3389/fbioe.2026.1929107)
Supplement: Supplementary file 1 [file Supplementaryfile1.docx]

Supplementary Material

# Supplementary Figures

**Supplementary Figure S1.** Air temperature (°C, red), relative humidity (%, pink), and rainfall (mm, blue) recorded from field trials: (A) Santa Cruz do Rio Pardo, SP (experiment 2), from July 2018 to July 2020; and (B) Ibaté, SP (experiments 1, 3, and 6), from July 2019 to July 2023.

**1 cm**

**A**

**B**

**Supplementary Figure S2.** Leaves showing (A) trifoliolate and (B) monofoliate phenotypes from seedlings at 120 days after sowing of open-pollinated Clemenules clementine progeny from experiment 3.

## Supplementary Tables

**Supplementary Table S1.** Information from field trials with genetically modified sweet orange trees: size of the area, number of plants, and geographical coordinates.

| **Experiment** | **Area size (hectares)** | **Plants (GM/non-GM)** | **Geographical coordinates** | |
| --- | --- | --- | --- | --- |
|  |  |  | **X** | **Y** |
| 1 | 3.04 | 2025 (650/1375) | 796252.0006 | 7565209.5470 |
| 2 | 4.19 | 2790 (1590/1200) | 46793.2619 | 7467137.5344 |
| 3 | 2.90 | 1932 (1156/776) | 796489.8710 | 7565047.7832 |
| 6 | 0.56 | 375 (165/210) | 796456.8744 | 7565205.4770 |

**Supplementary Table S2.** Cumulative plant eradication data in experiments 1, 2, 3, and 6 in the last year of the experiments (2023).

| **Location** | **Genotype** | **Number of planted trees (n)** | **Eradicated trees** | |
| --- | --- | --- | --- | --- |
|  |  |  | **n^a^** | **%** |
| **Experiment 1** |  |  |  |  |
| Border | Citrandarin | 334 | 1 | 0.3 |
|  | Clemenules | 350 | 58 | 16.6 |
|  | Pera sweet orange | 366 | 201 | 54.9 |
|  | **Total** | **1,050** | **260** | **24.8** |
| Parcels | Clemenules | 15 | 3 | 20.0 |
|  | Sour orange | 50 | 2 | 4.0 |
|  | Tahiti lime | 50 | 18 | 36.0 |
|  | Navelina sweet orange | 65 | 48 | 73.8 |
|  | Natal sweet orange | 65 | 17 | 26.2 |
|  | Pera sweet orange | 15 | 7 | 46.7 |
|  | Pineapple sweet orange | 390 | 78 | 20.0 |
|  | **Total** | **650** | **173** | **26.6** |
| **Experiment 2*** |  |  |  |  |
| Border | Citrandarin | 348 | 4 | 1.1 |
|  | Clementine | 380 | 80 | 21.1 |
|  | Pera sweet orange | 412 | 108 | 26.2 |
|  | **Total** | **1,140** | **192** | **16.8** |
| Parcels | Clemenules | 60 | 8 | 13.3 |
|  | Sour orange | 40 | 3 | 7.5 |
|  | Tahiti Lime | 70 | 21 | 30.0 |
|  | Navelina sweet orange | 390 | 72 | 18.5 |
|  | Natal sweet orange | 65 | 11 | 16.9 |
|  | Valencia sweet orange | 100 | 0 | 0.0 |
|  | Pera sweet orange | 160 | 3 | 1.9 |
|  | Pineapple sweet orange | 790 | 85 | 10.8 |
|  | **Total** | **1,675** | **203** | **12.1** |
| **Experiment 3** |  |  |  |  |
| Border | Citrandarin | 414 | 3 | 0.7 |
|  | Clemenules | 430 | 61 | 14.2 |
|  | Pera sweet orange | 446 | 207 | 46.4 |
|  | **Total** | **1,290** | **271** | **21.0** |
| Parcels | Pera sweet orange | 520 | 51 | 9.8 |
|  | Pineapple sweet orange | 65 | 11 | 16.9 |
|  | Valencia sweet orange | 650 | 141 | 21.7 |
|  | **Total** | **1,235** | **203** | **16.4** |
| **Experiment 6** |  |  |  |  |
| Border | Citrandarin | 60 | 0 | 0.0 |
|  | Clemenules | 60 | 9 | 15.0 |
|  | Pera sweet orange | 60 | 8 | 13.3 |
|  | **Total** | **180** | **17** | **9.4** |
| Parcels | Pera sweet orange | 120 | 5 | 4.2 |
|  | Valencia sweet orange | 75 | 3 | 4.0 |
|  | **Total** | **195** | **8** | **4.1** |

(a) Total of eradicated plants per genotype and location (parcels or border).

* Experiment 2 ended in July 2021, when plants were eradicated.

**Supplementary Table S3.** Primers used for amplification of the neomycin phosphotransferase type II (*npt*II) from DNA extracted from seedlings to assess transgene flow.

| **Primer** | **Sequence (5’ to 3’)** | **Reference** |
| --- | --- | --- |
|  |  |  |
| nptII-F | GACGAGGCAGCGCGGCTAT | Ghorbel et al., 2000. |
| nptII-R | AAGAAGGCGATAGAAGGCGA |  |

**Supplementary Table S4.** Paternity assignment analysis using phenotypic data (trifoliate and monofoliate leaves) and microsatellite markers (for monofoliate leaves) in the Clemenules clementine progeny from controlled pollinations during the 2011/2012 season.

| **Pollen grain treatment ^1^** | **Assigned paternity** | | | |
| --- | --- | --- | --- | --- |
|  | **Citrandarin ^2^** | **Sweet orange** | **Undetermined** | **Total** |
| Citrandarin 1600 | 284 (99.0%) | 1 (0.3%) | 2 (0.7%) | 287 (100%) |
| Citrandarin 1600 + Pineapple sweet orange | 414 (68.6%) | 153 (25.4%) | 36 (6.0%) | 603 (100%) |
| Citrandarin 1600 + Pera sweet orange | 322 (78.5%) | 59 (14.4%) | 29 (7.1%) | 410 (100%) |

^1^ – pollen grains we collected, dried, counted, stored and used in single pollinations or mixed pollinations (23).

^2^ – number represent the number of seedling samples assessed and number in brackets is the percentage.

Supplementary Table S5. Sampling data on Clemenules border plants: sampled plants, fruits collected, seeds extracted, and seeds per fruit.

| **Experiment** | **Season** | **Sampled plants** | **Fruits** | **Seeds** | **Seeds per fruit** |
| --- | --- | --- | --- | --- | --- |
| **1** | 2017/2018 | 60 | 2,839 | 9,189 | 3.3 ± 1.3 |
|  | 2018/2019 | 60 | 5,843 | 10,759 | 1.8 ± 1.2 |
|  | 2019/2020 | 47 | 2,157 | 2,313 | 1.1 ± 0.8 |
|  | 2020/2021 | 45 | 2,250 | 4,925 | 2.1 ± 1.5 |
| **2** | 2018/2019 | 56 | 5,542 | 3,261 | 0.6 ± 0.2 |
|  | 2019/2020 | 55 | 2,145 | 6,315 | 2.9 ± 1.2 |
| **3** | 2019/2020 | 120 | 2,816 | 3,857 | 1.4 ± 0.5 |
|  | 2020/2021 | 103 | 2,492 | 3,263 | 1.3 ± 0.4 |
|  | 2021/2022 | 87 | 2,499 | 5,257 | 2.5 ± 1.5 |
| **6** | 2019/2020 | 6 | 136 | 144 | 1.0 ± 0.4 |
|  | 2020/2021 | 6 | 150 | 230 | 1.5 ± 0.6 |
|  | 2021/2022 | 5 | 125 | 267 | 2.2 ± 0.7 |

**Supplementary Table S6.** Data from Pera sweet orange border plants in experiment 3: number of seeds, germinated seedlings per seed, and polyembryony.

| **Border** | **Season** | | **Seeds** | **Seedlings** | **Seedlings per seed** | | | | | |  | | **Polyembryony** | | |
| --- | --- | --- | --- | --- | --- | --- | --- | --- | --- | --- | --- | --- | --- | --- | --- |
|  |  |  |  |  | **1** | **2** | **3** | **4** | **5** |  | | **n ^a^** | | **%** |  |
| Internal | 2021/22 | | 200 | 319 | 101 | 83 | 15 | 0 | 1 |  | | 100 | | 49.50 |  |
|  | 2022/23 | | 1,599 | 1,947 | 1,311 | 230 | 56 | 2 | 0 |  | | 288 | | 18.01 |  |
| External | 2021/22 | | 650 | 959 | 393 | 210 | 41 | 6 | 0 |  | | 257 | | 39.54 |  |
|  | 2022/23 | | 547 | 451 | 432 | 98 | 11 | 5 | 1 |  | | 115 | | 21.02 |  |
| Sum/Average | |  | 2,996 | 3,676 | 2,237 | 621 | 123 | 13 | 2 |  | | 760 | | 32.02 |  |

^a^ Total of polyembryonic seeds.

**Supplementary Table S7.** Sampling data on Clemenules control plants: sampled plants, fruits collected, seeds extracted, and seeds per fruit.

| **Experiment** | **Season** | **Sampled plants*** | **Fruits** | **Seeds** | **Seeds per fruit** |
| --- | --- | --- | --- | --- | --- |
| **1** | 2017/18 | 15 | 1,142 | 4,506 | 3.8 ± 1.2 |
|  | 2018/19 | 13 | 1,289 | 1,272 | 1.0 ± 0.3 |
|  | 2019/20 | 15 | 725 | 530 | 0.7 ± 0.2 |
|  | 2020/21 | 15 | 750 | 1,098 | 1.5 ± 0.3 |
| **2** | 2018/19 | 24 | 2,141 | 1,699 | 0.8 ± 0.3 |
|  | 2019/20 | 24 | 952 | 3,430 | 3.6 ± 0.6 |

* Plants with available fruit for analysis (Experiment 1, season 2018/2019 had only 13 out of 15 plants sampled).
